# A Preliminary Parasitological Survey of *Hepatozoon* Spp. Infection in Dogs in Mashhad, Iran

**Published:** 2012

**Authors:** AA Rahmani Amoli, J Khoshnegah, GhR Razmi

**Affiliations:** 1 Department of Clinical Sciences, Faculty of Veterinary Medicine, Ferdowsi University of Mashhad, Mashhad, Iran; 2 Department of Pathobiology, Faculty of Veterinary Medicine, Ferdowsi University of Mashhad, Mashhad, Iran

**Keywords:** *Hepatozoon*, Prevalence, Blood, Parasitology, Dog, Iran

## Abstract

**Background:**

We attempted to determine the prevalence of *Hepatozoon* spp. infection in Mashhad, northeast of Iran, via blood smear parasitology.

**Methods:**

The prevalence was investigated by examination of blood smear parasitology, using blood samples collected from 254 dogs (51 strays and 203 privately owned-dogs).

**Results:**

Two stray dogs (2/51; 3.92%) and two privately-owned dogs (2/203; 0.98%) were infected with *Hepatozoon* spp. Therefore, as per blood smear parasitology, the prevalence of *Hepatozoon* spp. infection was 1.57% (4/254). Sixteen out of 254 dogs (6.29%) were infested with ticks; all of which were *Rhipicephalus sanguineus*. One of the dogs infected with *Hepatozoon* spp. exhibited ticks at the time of examination. Concurrent infection with *Ehrlichia canis* and *Leishmania infantum* was not detected in the four *Hepatozoon* spp. infected dogs.

**Conclusion:**

This is the first epidemiological study on the prevalence of *Hepatozoon* spp. infection in dogs in Iran.

## Introduction

*Hepatozoon* is a tick borne protozoan parasite, classified in the Phylum Apicomplexa and is closely related to *Plasmodium* spp. and *Piroplasms* (1). The parasite is primarily transmitted by the brown dog tick, *Rhipicephalus sanguineus* via the ingestion of ticks, or parts of ticks containing mature *Hepatozoon* spp. oocysts (2). In dogs, the infection results in the development of schizonts within various tissues and gamonts in the peripheral blood (3–5).

*Hepatozoon* spp. infection produces variable manifestations. The infection can result in an asymptomatic state, with low parasitemia, or can culminate into a severe, life-threatening illness, resulting in fever, lethargy, anemia, and emaciation, with high levels of parasitemia (6). Hepatozoonosis is mainly diagnosed by the observation of intracellular *Hepatozoon* gamonts within neutrophils in Giemsa-stained peripheral blood smears (7). Although canine hepatozoonosis was first reported in India in 1905 (8), it has only recently been noted in Iran (9).

The epidemiology of *Hepatozoon* spp. infection remains to be established and, thus, this preliminary epidemiological study was attempted to determine the prevalence of *Hepatozoon* spp. infection in the Mashhad, northeast of Iran, via blood smear parasitology.

## Materials and Methods

### Dogs and blood smear parasitology

The sampling was performed from June 2010 to July 2011. Blood samples were collected from 51 stray dogs, captured and kept in the municipality shelter located in Mashhad, Iran. In addition, 203 privately owned-dogs were admitted to the Ferdowsi University of Mashhad Veterinary Teaching Hospital and were screened for the presence of *Hepatozoon* spp. All the dogs were examined for the presence of fever, depression, anorexia, weight loss, oculonasal discharge, lymphadenopathy, skin and hair coat changes and membranous pallor. Blood samples (5 ml) were drawn from the cephalic vein of each dog and were placed into plain and Ethylenediaminetetraacetic acid (EDTA) tubes. A thin blood smear was prepared for each sample, fixed with methanol and stained with Giemsa. Complete blood counts were performed manually for all dogs, and the presence of hematological disorders was recorded for each animal. The parasitemia was calculated manually by counting 500 neutrophils under oil immersion field (14).

This research proposal has received ethical approval by Ferdowsi University of Mashhad Research Office.

### Diagnosis of concurrent infections

The sera were separated off and frozen at 20 °C for serological assays. All sera were examined for the presence of the antibodies against *Ehrlichia canis* and *Leishmania infantum* by indirect immunofluorescence antibody (IFA) test kit (Mega Screen^®^ FLUO; MegaCor, Horbranz, Austria) in accordance with the manufacture's recommendations. The stained slides were read under 400 magnification fluorescence microscope (Olympus, BH-2). Evaluation was carried out with green and red filter.

## Results

### Blood smear parasitology

**Fig. 1:**
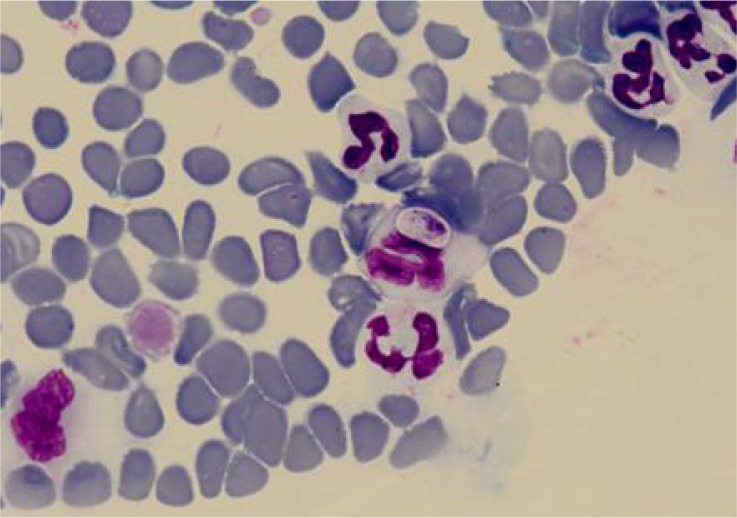
Gametocyte of *Hepatozoon* spp. in a neutrophil from peripheral blood smear (Giemsa's stain ×1,000)

Blood smear parasitology, revealed 1.57% (4/254) of the subjects were infected by *Hepatozoon* spp. (Fig. 1). These included two stray (2/51; 3.92%) and two privately-owned dogs (2/203; 0.98%). The parasitemia ranged from 1 to 3% with the highest parasitemia (3%) found in a 15-year-old male privately-owned dog.

### Tick infestation

Out of 254 dogs, 16 (6.29%) were infested with ticks; all of which were *R. sanguineus*. The ticks were found at the surface of the skin on one of the four infected dogs.

### Clinical and hematological findings

Anorexia was observed in two infected dogs. Anemia was detected in 67 of the 254 dogs (26.37%), two of which were *Hepatozoon* spp. infected. Table 1 summarizes the clinical and hematological results from the four *Hepatozoon* spp. infected dogs.

**Table 1:** Clinical and hematological findings of the four dogs infected with *Hepatozoon* sp.

Dog No.	Breed	Age (yr)	Sex	Clinical sign	Tick infestation	Parasitemia(%)	PCV%	Total WBC (/µL)	Neutrophils (/µL)	TP (g/dL)
1	Undetermined	15	M	Anorexia, superficial pyoderma	+	3	52	7500	4950	7
2	Terrier	1. 5	F	-	-	1	34	7000	4060	6.8
3	Mixed breed	3	M	Broken hind limb	-	1	41	10150	7105	6.1
4	Mixed breed	3	M	Anorexia, pale mucous membranes and alopecia	-	1	37	11000	9020	7.9

Reference values (24) (Willard and Tvedten 2004); PCV: 37-55, Total WBC (/µL):6000-17000, Neutrophils (/µL): 3000-11500

### Concurrent infections

Twenty-two of 254 (8.67%) serum samples had antibodies to *L. infantum*. Also, a very low prevalence of anti-*E*. *canis* antibodies (0.8%; 2/254), was detected among studied dogs (unpublished data). In the four *Hepatozoon* spp. infected dogs, concurrent infection with these pathogens were not detected.

## Discussion

In this preliminary epidemiological survey, we attempted to reveal the prevalence of *Hepatozoon* spp. infection among dogs living in Mashhad, northeast of Iran. As per this study, the prevalence of *Hepatozoon* spp. infection was 1.57% by examination of blood smear parasitology. In contrast to the present study, others have reported a rather high prevalence of *Hepatozoon* spp. infection. Garcia de Sá et al. examined 31 dogs from rural areas and identified 7 dogs (22.6%) positive by blood smear examination (10). Rubini et al. reported a prevalence of 11.3% (17/150) using Giemsa-stained blood smears (11).

In the present study, we used peripheral blood smears to detect *Hepatozoon* spp. gamonts. Buffy coat smears, however, may have significantly increased the sensitivity of the detection of *Hepatozoon* spp. gamonts compared to peripheral blood smears as shown previously for the detection *E*. *canis* morulae by microscopy (12). There is also evidence that direct observation is less sensitive than IFAT since the prevalence of *H. canis* infection investigated by blood smear parasitology was only 1%, as compared to IFAT yielding a 33.1% finding (13). Karagenc et al. showed that the number of *Hepatozoon* positive animals detected by IFAT were significantly higher than that determined by PCR and microscopy (14). The absence of gamonts in IFAT positive animals may be due to low or intermittent parasitemia or arrest of parasite development at the meront stage in visceral organs (6, 13, 15). Alternatively, anti-*Hepatozoon* spp. antibodies may have persisted for months after parasitemia can no longer be detected (16, 17).

In the present study, one of the *Hepatozoon* spp. infected dogs was old (15 yr) and the other three were young (1.5 to 3 yr). *Hepatozoon* spp. infection was found in dogs from 9 months to 7 years old (18). *Hepatozoon* spp. infection was the most prevalent in dogs less than 6 months and in dogs 5 to 10 years old (6). Similar to the present study, it has also been reported that *Hepatozoon* spp. infection can be seen in both sexes (18).The distribution of *Hepatozoon* is tied closely to its primary vector, *R. sanguineus* (2). This tick is considered as abundant tick species in northeast of Iran (19).

In the present study, the infected dogs were asymptomatic, with low parasitemia, ranged from 1% to 3%. A low level of *Hepatozoon* spp. parasitemia with gamonts found in less than 5% of neutrophils is the most common form of infection and is usually associated with an asymptomatic or mild disease. A high level of parasitemia, however, would be associated with significant clinical signs (3, 6). The present study indicates that the exposure to *Hepatozoon* spp. was very low amongst the dogs, in our area. In addition, it appears that most of the infected dogs were asymptomatic and only a relatively small number of animals (12.2%), developed the severe form of the disease. Previously, Khoshnegah et al. described a severe form of hepatozoonosis occurred in an 11-year-old male dog. Clinical signs included anorexia, weight loss, depression, nasal and ocular discharge, in coordination of the posterior limbs, a mildly painful hind limb, peripheral lymphadenopathy, pale mucous membranes but afebrile (9). In cases where gamonts are detected upon blood smear parasitology, the various clinical signs exhibited by the infected dogs are attributed to concurrent infections due to other, more potent pathogens such as *Babesia canis*, *Ehrlichia* spp., *Leishmania*, etc (20–23). In the present study, concurrent infection with other pathogens (including *E. canis* and *L. infantum*) were not detected in the four *Hepatozoon* spp. infected dogs. There is also the possibility that an interaction between the different pathogens might exist, which in turn might lead to further deterioration in the infected dogs’ clinical condition (6).

## Conclusion

The present study demonstrates a low prevalence of *Hepatozoon* spp. infection among dogs residing in the northeast of Iran. One limitation of the direct observation is that gametocytes are not always detectable since parasitemia may be intermittent or the number of circulating gametocytes very low. From a clinical perspective, practitioners should be aware of *Hepatozoon* spp. and should include its screening in their routine diagnostic panel. Misdiagnosis may lead to inappropriate treatment and relapses. As soon as the disease is diagnosed, practitioners should know that since elimination of gametocytes from the peripheral blood is slow, an 8-week treatment is always required (1). Canine hepatozoonosis in Iran may be caused by *H. canis* or by a new species of *Hepatozoon* and could be considered endemic. Further work based on PCR and serological tests is necessary to confirm the species causing this disease in Iran. Most importantly, strict control measures against ticks applied in dog kennels and shelters may prevent the spread of tick-borne diseases.
